# The First Year of the New Century

**Published:** 1901-12

**Authors:** 


### Editorial.

### THE FIRST YEAR OF THE NEW CENTURY.

It is always interesting to the casuist to note the effect of certain periods upon the mental and moral forces, whether this be the beginning of a month, a year, or a century, and upon this rather weak foundation a prophecy is based from the periods named for the future. In some respects this certainly rises above mere conjecture and assumes the characteristic of true scientific deduction.

The human mind is liable to sudden changes, both in the individual and in the mass; and while these impulsively run from low to high and in reverse order, there is nevertheless a certain stability, a steady advance towards a higher state. If this were not so, the modern civilization with which we are familiar would have been impossible. This constitutes the hope of the altruist for the future, for to his view the time will surely come when the barbarism of the present, with its savage wars and apologists for brutalities, will have passed away through these positive and uplifting tendencies forcing the human mind eventually to a higher moral standard.

When, therefore, the new era, the beginning of another century, is upon us, the change, while arbitrary, naturally leads to reflection, and that of the first year of the century to a critical comparison with the one hundred years of the preceding century. The history of that period is assured. The work of the wonderful nineteenth century is behind us, and will the twentieth uphold that reputation and pass on to better things? The first year, of which this is the last month, must give some indication of this if existent, and upon this we may draw, in part, the horoscope of the next one hundred years.

This first year has found the world in somewhat of a chaotic condition. Transition periods are always periods of suffering and discouragement. The civilizations of the world have been on trial, and the ethics of the past have been found wanting. It may be too much to say that there has been no real advancement upon the moral side, but the evidence seems to force to that conclusion. Empires and republics are alike preparing for the eventual conflict which the new century is sure to witness. Until this is over, the reign of peace, which the moral enthusiast longs for, will fail to come. After this, arbitration between nations.

It is impossible, while taking a cursory observation of all the conflicting interests of the world, not to reach a definite conclusion that through all this upheaval there is an undercurrent of pure motives that will eventually attain a higher standard in ethics as inevitably as that the earth and its atmosphere tend more and more to refinement, the physical and moral naturally correlated with each other.

Our interest is not so much with the advance of mind generally, important as that is, but whether the signs visible in the first year of the century portend anything favorable. A year is but a limited time, and yet it means much if in that twelve months even one single advance be made.

The review cannot be confined to individuals. They are but the units of the organization. Upon their health will depend that of the body as a whole; but as these cannot be examined individually, judgment must be formed by a consideration of the mass.

If then, in dentistry, we examine the concrete as represented by the various local, State, and national bodies, we have reason to infer that the individual is growing; in fact, no recent year has done so much in the direction of progress. The national bodies have exhibited much better work in this country during that brief period. The international organizations have brought the labor of the dental world into closer and more harmonious relations. Who could have imagined one year ago that sixteen nationalities could possibly have been brought together in London to discuss a more harmonious arrangement of dental education? Nothing that has occurred this past year of a more encouraging nature than this, even though the final result may not be all that is hoped for. It is the beginning of the destruction of those international jealousies that have done so much to embitter and embroil nations and keep apart members of the same profession. Those who have not read the able and exceedingly clear-sighted address of Sir Michael Foster before the International Dental Federation, and published in our last number, should not fail to do so. It marks a very advanced step in human as well as professional progress worthy the man and indicative of the new era rapidly approaching.

Who can fail to see in the almost unanimous action of the Association of Faculties, in advancing the course in all the dental colleges to four years, to be another sign of positive growth?

What means the activity of the local organizations, striving with each other as never before to eliminate the crudities of the past and in their place substitute more of real science?

Who cannot see in the active prosecution and the crushing out of the fraudulent diploma establishments a healthy sign that the apathy of the past is over, and that the new year points to a professional body that eventually will be full of vigorous moral health?

While all these outward signs are most encouraging, there seems to be an inward, but not a prominent one, indicative of a tendency to a broad conception of duty and practice. The narrowness—the result of isolation—will doubtless continue; but as the years go by the experiences of this one year will have become fixed as moral and professional facts, and those who live to see it will find the dental profession has risen equal to its high duties and responsibilities, and that the prophetic foundation of the first year of the century has not been laid in vain.

